# Reactive oxygen species scavenging and inflammation mitigation enabled by biomimetic prussian blue analogues boycott atherosclerosis

**DOI:** 10.1186/s12951-021-00897-2

**Published:** 2021-05-31

**Authors:** Yan Zhang, Yifei Yin, Wei Zhang, Hongyan Li, Taixia Wang, Haohao Yin, Liping Sun, Chunxia Su, Kun Zhang, Huixiong Xu

**Affiliations:** 1 Department of Medical Ultrasound and Central Laboratory, Shanghai Tenth People’s Hospital, Ultrasound Research and Education Institute, Clinical Research Center for Interventional Medicine, Tongji University School of Medicine, Shanghai Engineering Research Center of Ultrasound Diagnosis and Treatment, National Clinical Research Center for Interventional Medicine, No. 301 Yan-chang-zhong Road, Shanghai, 200072 People’s Republic of China; 2 Department of Radiology, Affiliated Hospital of Guilin Medical University, No. 15 Le-Qun Road, Xiufeng District, Guilin, 541001 Guangxi People’s Republic of China; 3 Department of Medical Oncology, Shanghai Pulmonary Hospital, Thoracic Cancer Institute, Tongji University School of Medicine, No. 507 Zheng-Min Road, Shanghai, 200433 People’s Republic of China

**Keywords:** Atherosclerosis, Nanomedicine, Oxidation stress, Anti-inflammation, Prussian blue analogue

## Abstract

**Background:**

As one typical cardiovascular disease, atherosclerosis severely endanger people’ life and cause burden to people health and mentality. It has been extensively accepted that oxidative stress and inflammation closely correlate with the evolution of atherosclerotic plaques, and they directly participate in all stages of atherosclerosis. Regarding this, anti-oxidation or anti-inflammation drugs were developed to enable anti-oxidative therapy and anti-inflammation therapy against atherosclerosis. However, current drugs failed to meet clinical demands.

**Methods:**

Nanomedicine and nanotechnology hold great potential in addressing the issue. In this report, we engineered a simvastatin (Sim)-loaded theranostic agent based on porous manganese-substituted prussian blue (PMPB) analogues. The biomimetic PMPB carrier could scavenge ROS and mitigate inflammation in vitro and in vivo. Especially after combining with Sim, the composite Sim@PMPB NC was expected to regulate the processes of atherosclerosis. As well, Mn^2+^ release from PMPB was expected to enhance MRI.

**Results:**

The composite Sim@PMPB NC performed the best in regulating the hallmarks of atherosclerosis with above twofold decreases, typically such as oxidative stress, macrophage infiltration, plaque density, LDL internalization, fibrous cap thickness and foam cell birth, etc*.* Moreover, H_2_O_2_-induced Mn^2+^ release from PMPB NC in atherosclerotic inflammation could enhance MRI for visualizing plaques. Moreover, Sim@PMPB exhibited high biocompatibility according to references and experimental results.

**Conclusions:**

The biomimetic Sim@PMPB theranostic agent successfully stabilized atherosclerotic plaques and alleviated atherosclerosis, and also localized and magnified atherosclerosis, which enabled the monitoring of H_2_O_2_-associated atherosclerosis evolution after treatment. As well, Sim@PMPB was biocompatible, thus holding great potential in clinical translation for treating atherosclerosis.

**Graphic abstract:**

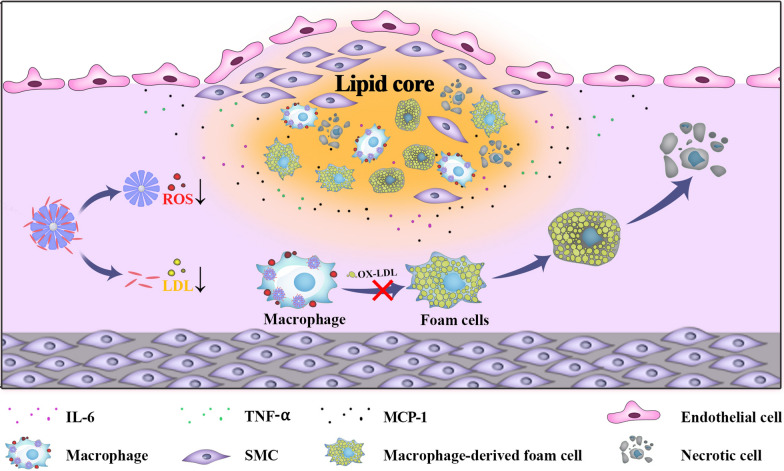

**Supplementary Information:**

The online version contains supplementary material available at 10.1186/s12951-021-00897-2.

## Introduction

Cardiovascular diseases are highly prevalent and cause the most deaths across the whole world, approximately accounting for nearly half of all deaths [1]. Atherosclerosis on arterial wall is the primary driver of cardiovascular diseases, but can be directly switched into acute clinical events (*e.g.*, sudden cardiac death) once plaque rupture and accompanied thrombosis occur [2] which, thus, imposes a substantial healthcare burden on patients [3]. Starting from endothelial layer dysfunction, atherosclerotic plaques as the hallmark of atherosclerosis are progressively evolved over time. In this evolution process, some typically pathological characteristics were concurrently discerned in the intimal layer, *e.g.*, oxidized low-density lipoproteins (LDL) accumulation, systematic and local inflammation, oxidative stress augmentation, macrophage infiltrations, collagen-rich fibrous cap development and foam cell birth, etc*.* [4, 5].

In particular, comprehensively understanding the pathogenesis of atherosclerosis and figuring out the influences of some processes (*e.g.*, lipoprotein oxidation, inflammation and oxidative stress) on atherosclerotic plaque formation will provide in-depth insights into atherosclerosis and benefit new treatment means’ birth [6]. Typically, inflammation and excessive oxidative stress have been demonstrated to closely correlate with the development and progression of atherosclerosis [7–11], and directly participate in all stages of atherosclerosis [12–14]. In detail, due to the imbalance between oxidant and anti-oxidant actions in vascular injures, uncontrolled ROS production will induce excessive oxidative stress to oxidize LDL. The oxidized LDL is easily accumulated in the intimal layer to promote local inflammation, which further induces a series of structural and physiological changes [7, 11, 15]. Based on this fact, inflammation and oxidative stress that have been regarded as the hallmarkers of atherosclerosis can serve as the targets to stimulate the development of various therapeutic drugs and predict cardiovascular events with acute coronary syndromes [15–17]. Concomitantly, anti-oxidative therapy and anti-inflammation therapy were developed [15].

However, currently available antioxidants fail to meet clinical demands to some extent. Fortunately, the tremendous advances in nanomedicine are expected to furnish rich tools to tackle them [18–20]. Typically, some targeted theranostic agents capable of scavenging oxidation stress were demonstrated to eliminate ROS and resist inflammation [10]. More intriguingly, some iron oxide nanoparticles (IONs)-involved theranostic nanoplatforms were equipped with the abilities to targetedly localize and visualize angiogenesis and supervise atherosclerosis-associated inflammatory changes in the vessel wall [21–23]. However, the current investigation on nanomedicine-enabled ROS scavenging is still at its infancy.

**Scheme1:**
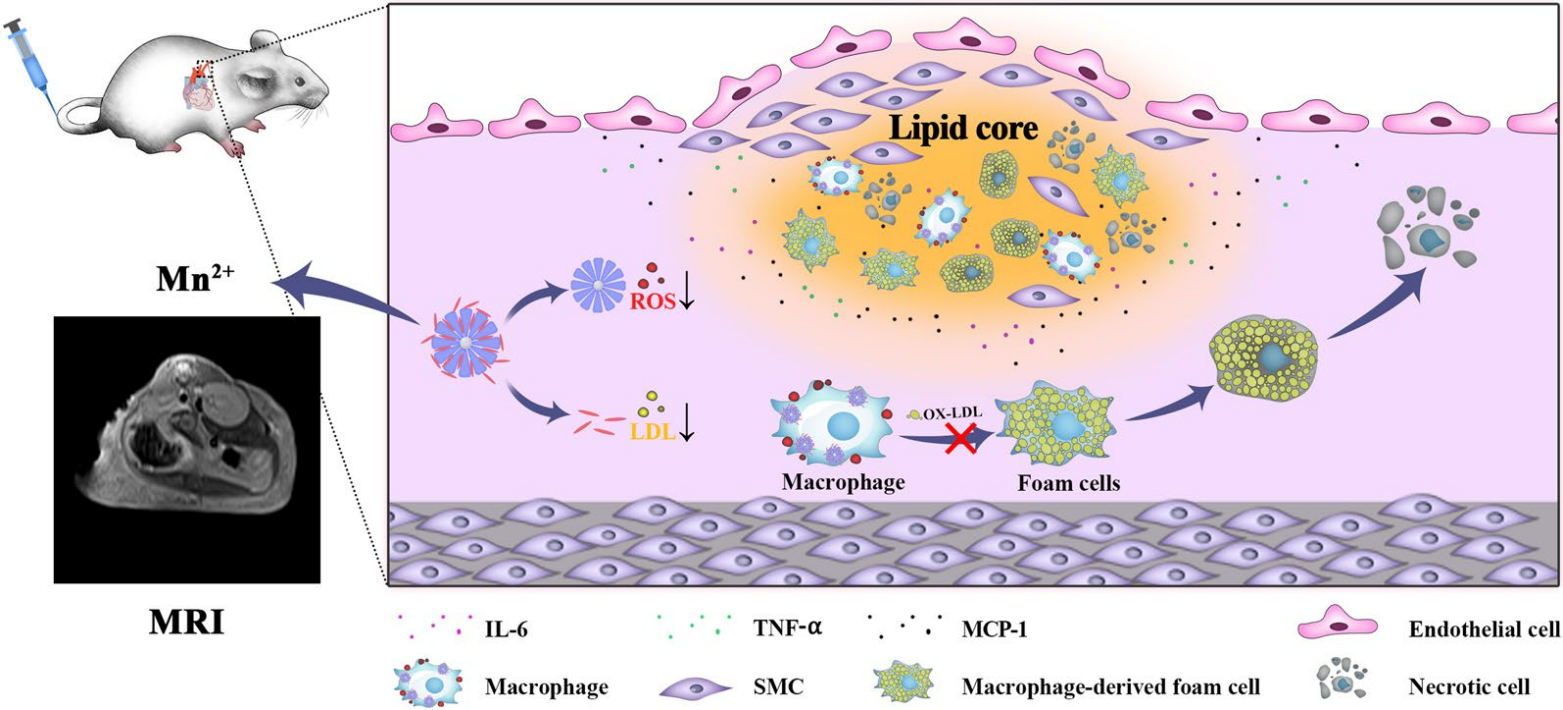
Schematic illustration of the underlying principle using Sim@PMPB NC to treat atherosclerosis

In this report, we used a biomimetic prussian blue (PB) analogues, *i.e.*, porous manganese-substituted prussian blue (PMPB) nanocubes (NC) to reduce atherosclerotic plaques and treat atherosclerosis. The PMPB NC could scavenge reactive oxygen species (ROS), mitigate inflammation, lower oxidized LDL internalization and hamper foam cell formation for boycotting atherosclerosis, as unraveled in Scheme 1. Akin to PB nanoparticles that favored inflammation alleviation in inflammatory bowel disease, ischemic brain damage, colitis, osteoarthritis, etc*.* [24–28] the biomimetic PMPB NC that served as enzyme-like catalysts were also allowed to exhaust ROS and lessen inflammation for treating atherosclerosis. To further potentiate the therapeutic outcome, a clinical anti-oxidant drug, *e.g.*, simvastatin (Sim), was loaded in PMPB NC due to the rich porosity of PMPB NC. Contributed by the two active components, the largest decreases of ROS level, pro-inflammatory cytokine secretion, collagen accumulation, fibrous cap thickness, macrophage infiltration, foam cell birth and LDL internalization were acquired, consequently depleting plaques and mitigating atherosclerosis. As well, Mn^2+^ release from PMPB after reaction with rich H_2_O_2_ in inflammation is expected [29, 30]. It determines that Mn^2+^-enhanced T1-MRI can be leveraged to localize and visualize atherosclerosis and monitor the H_2_O_2_-dependent treatment process, which is much preferable than Fe-enhanced T2-MRI that is usually insensitive to lesion microenvironment. More significantly, PBs have been approved in clinics by FDA [16, 31–33], which, along with aforementioned compelling results, drive clinical translation.

## Results and discussion

### Synthesis of biomimetic PMPB and Sim@PMPB NC

**Fig. 1:**
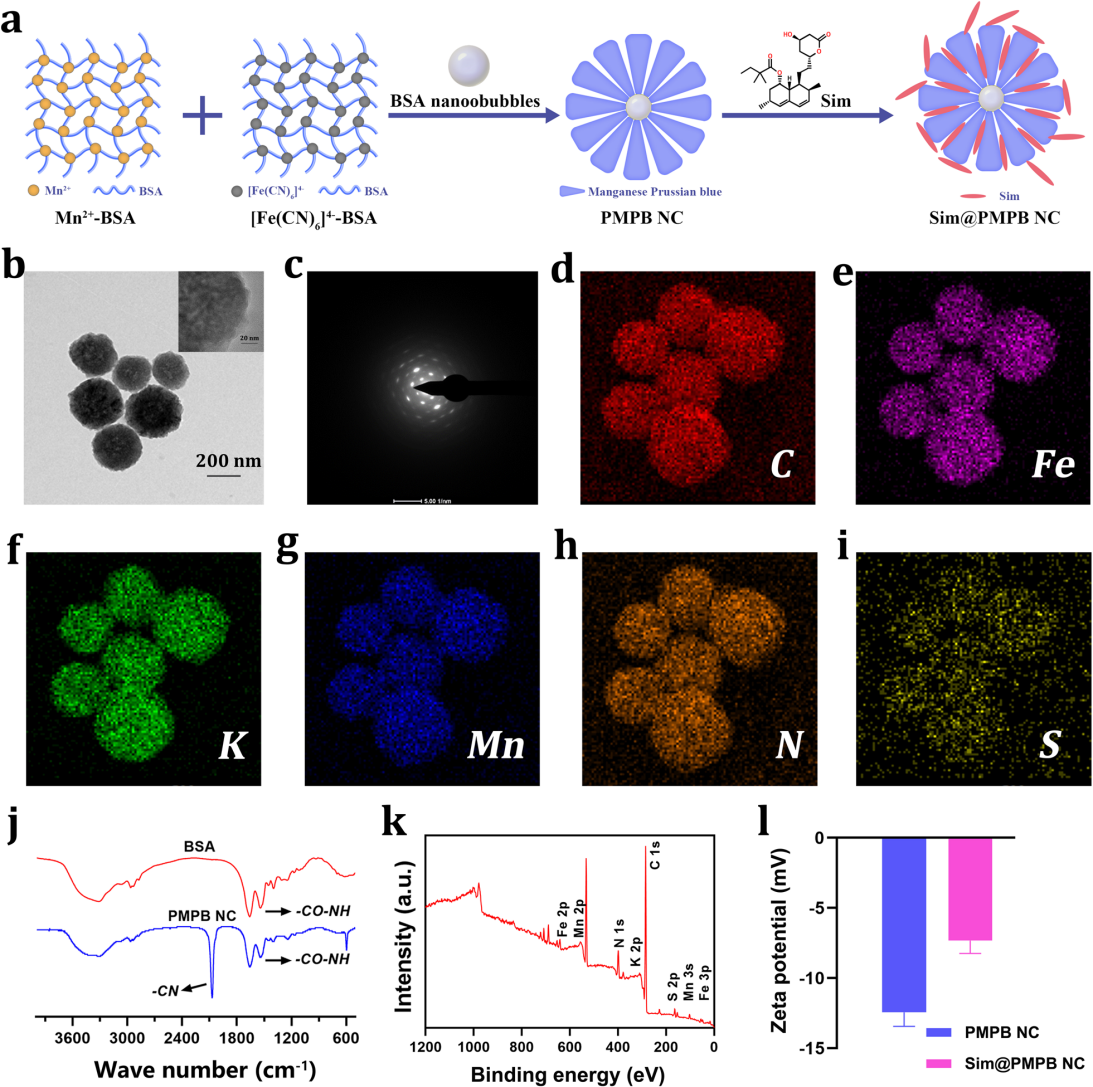
Synthesis and characterization of the biomimetic PMPB NC and Sim@PMPB NC. **a** Schematic illustration on the synthetic procedures of PMPB NC and Sim@PMPB NC. **b**, **c** TEM (**b**) and HRTEM (**c**) images of PMPB NC. **d** Electron diffraction pattern of PMPB NC within the selected region of interest. **d**–**i** The element mapping of PMPB NC, including C (**d**), Fe (**e**), K (**f**), Mn (**g**), N (**h**) and S (**i**). **j** FTIR spectra of BSA and PMPB NC. **f** XPS spectral of PMPB NC. **g** Zeta potentials of PMPB NC and Sim@PMPB NC that were obtained through electrophoretic light scattering (ELS) measurement on dynamic light scattering (DLS) equipment

The synthetic procedures of PMPB NC were shown in Fig. 1a, and they were obtained according to a classic reference [24]. Herein, bovine serum albumin (BSA) primarily serves as coordination chelators for Mn^2+^ and [Fe(CN)]^4−^ ions to promote Mn&Fe co-precipitation for yielding PMPB NC and it can also act as the template and pore-making agent. A well-dispersed PMPB NC with a diameter of 200 nm is observed (Fig. 1b and Additional file 1: Figure S1), and exhibits a high degree of crystallinity (Fig. 1c). Atom mapping test indicates the presence and uniform distribution of Fe, S, Mn, etc*.* (Fig. 1d–i), suggesting the successful synthesis of PMPB NC. As well, the typical representative peaks corresponding to -CN and -CO–NH in PMPB NC indicate the presence of BSA and reflect that PMPB NC is successfully obtained (Fig. 1j). X-ray photoelectron spectroscopy (XPS) was also used to verify it. Wide- and narrow-window XPS spectra clearly show the Mn and Fe atoms in PMPB NC (Fig. 1k and Additional file 1: Figure S2), and they are identical to previous reports that involved PB or Mn-doped PB [34–36].

**Fig. 2:**
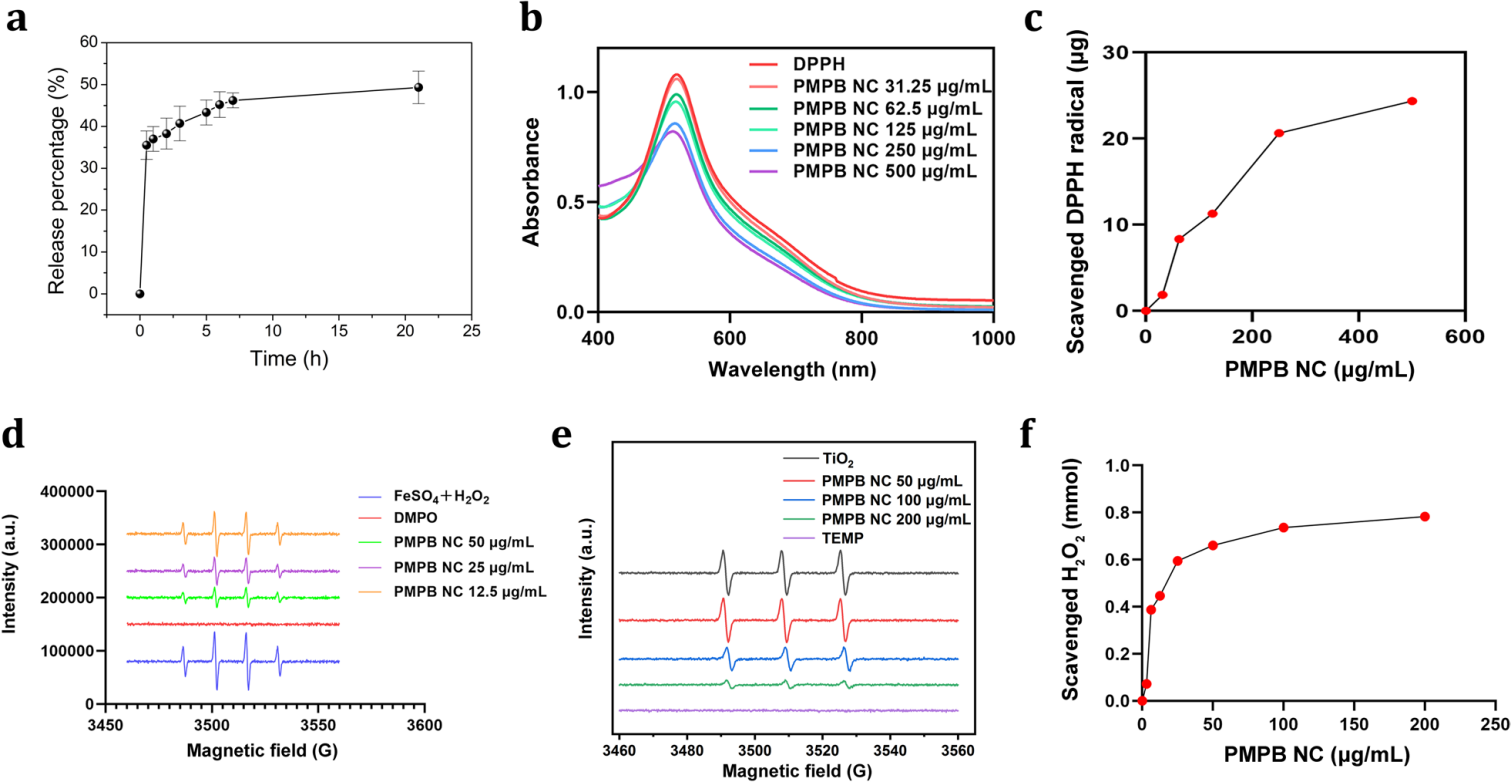
ROS-scavenging ability exploration of PMPB NC. (a) Time-dependent Sim release profile from Sim@PMPB NC. (b) UV–vis spectra of DPPH when incubating with PMPB NC with varied concentrations (500, 250, 125, 62.5, 31.25 and 0 μg/mL). (c) Dose-dependent elimination of DPPH by PMPB NC. (d) ESR spectra of different samples including the FeSO_4_ & H_2_O_2_ mixture and PMPB NC with varied concentrations (0, 50, 25, 12.5 μg/mL) dispersed in the mixture of FeSO_4_ and H_2_O_2_, where DMPO always existed in each group. (e) ESR spectra of different samples including PMPB NC with varied concentrations (0, 50, 100, 200 μg/mL) dispersed in the TiO_2_ dispersion in the presence of xenon light irradiation (300 W), where TEMP always existed in each group. (f) Dose-dependent elimination of H_2_O_2_ by PMPB NC

Thanks to the rich porosity and large pore diameter of PMPB NC (Additional file 1: Figures S1 and S3), Sim encapsulation is readily accessible to PMPB. UV–Vis spectra confirm the successful loading of Sim in PMPB NC (Additional file 1: Figure S4), and the loading amount of Sim is calculated to be approx. 3.84% according to the dose-absorbance standard curve of Sim (Additional file 1: Figure S5). Sim loading brings about the increase of zeta potential from − 12.4 to − 7.2 mV (Fig. 1l), and also cause the particle size to rise due to Sim adsorption on the surface of PMPB NC (Additional file 1: Figure S6). Sim adsorption on PMPB surface also favors high colloidal stability (Additional file 1: Figure S7). The loaded Sim is released in a slow but continuous manner (Fig. 2a).

### ROS scavenging test

High ROS scavenging ability is the premise of anti-inflammation and anti-oxidative therapy [10]. In an attempt to develop drugs featuring ROS scavenging, various anti-oxidant drugs were developed [15]. However, these drugs fail to guarantee high clinical efficacy. Herein, PMPB NC was expected to meet clinical demands of anti-oxidative therapy for atherosclerosis, especially after combining with the clinical anti-oxidative drug (Sim). To demonstrate it, the exhaustion ability of PMPB NC was scrutinized. An indirect test was firstly carried out, where the absorbance of 1,1-diphenyl-2-trinitrophenylhydrazine (DPPH) that is a indicator of ROS radicals was inspected after incubating with PMPB NC. UV–vis spectra show that the absorbance intensity of DPPH dye gradually drops as the concentration of PMPB NC (Fig. 2b). This compelling result suggests that PMPB indeed scavenged ROS radicals. Quantitative data also displays that PMPB NC exerts a robust total antioxidant effect through capturing and depleting ROS radicals (Fig. 2c) according to the standard curve of DPPH (Additional file 1: Figure S8).

Electron spin resonance (ESR) was further used to examine the scavenging ability of PMPB NC using 5,5-dimethyl-1-pyrroline N-oxide I (DMPO) as the capturing agent [37, 38]. Herein, the Fenton reaction between FeSO_4_ and H_2_O_2_ was adopted to give birth to free hydroxyl radicals (·OH) for assessing the scavenging ability of PMPB NC. It is clearly found that PMPB NC can wipe out the free radicals, and more PMPB NCs receive less residual radicals (Fig. 2d). As well, singlet oxygen scavenging by PMPB NC was also explored with (TEMP) as the capturing agent using ESR, wherein TiO_2_ was used to generate singlet oxygen in the presence of ultrasound irradiation. Inspiringly, the signal intensity gradually decays as the concentration of PMPB NC increases (Fig. 2e), suggesting PMPB NC also scavenged singlet oxygen that is another type of ROS. Furthermore, the H_2_O_2_ scavenging ability of PMPB NC was also explored since H_2_O_2_ is also a type of ROS [39]. Although H_2_O_2_ damage ability to artery vessels is weaker than free radicals, the much more H_2_O_2_ than free radicals in inflammation determines that artery vessel tissues are undoubtedly subjected to severe H_2_O_2_-induced damages [30]. Intriguingly, H_2_O_2_ is also cleared by PMPB NC, and more PMPB NCs trigger more H_2_O_2_ scavenging till to a saturation plateau, as evidenced in Fig. 2f.

### Cellular-level anti-oxidation and anti-inflammation explorations

**Fig. 3:**
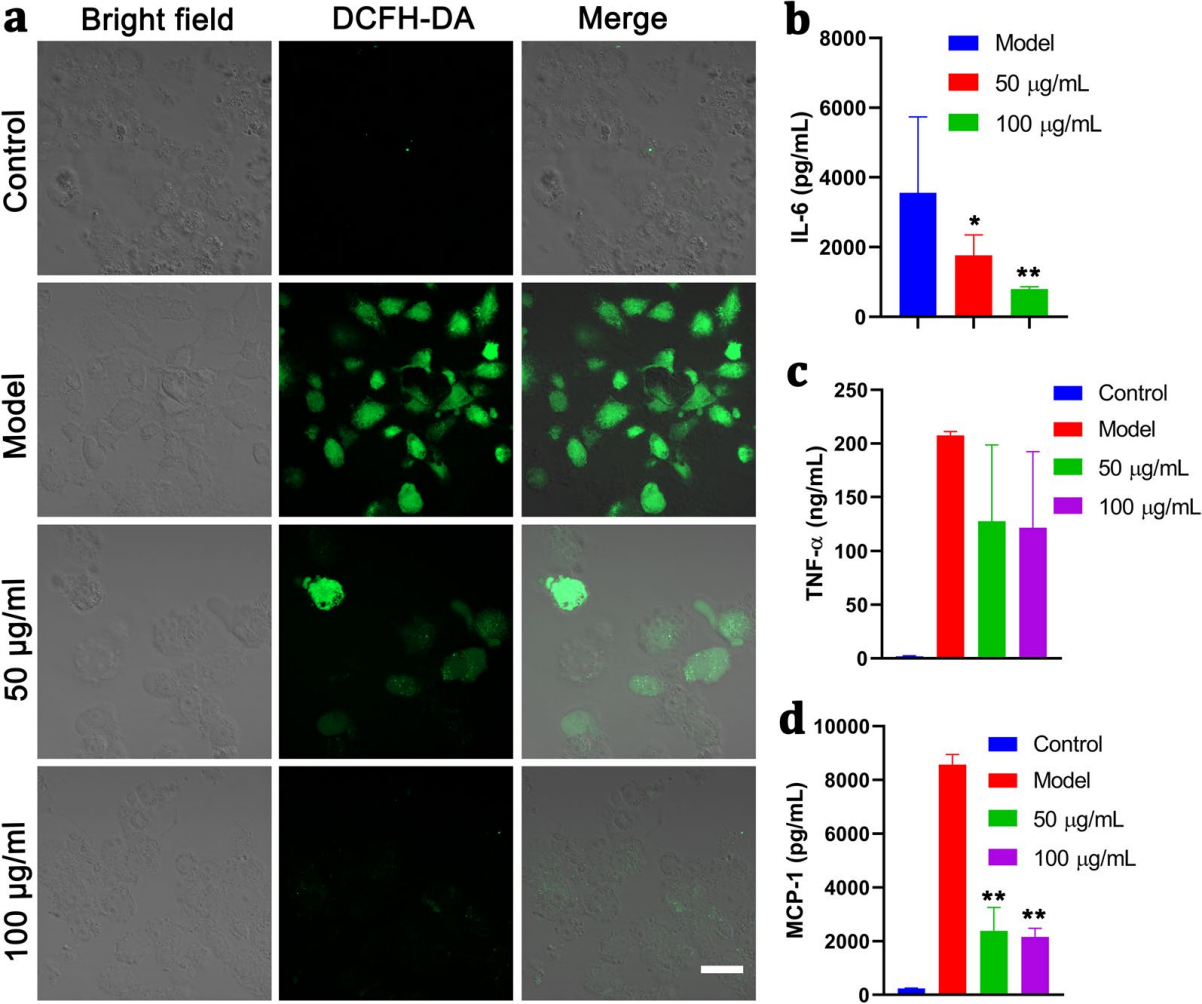
In vitro anti-oxidation and anti-inflammation using PMPB NC via scavenging ROS. **a** Confocal fluorescence images of RAW264.7 cells after different corresponding treatments in four groups, *i.e.*, control, Model, 50 μg/mL, 100 μg/mL), where ROS indicator, *i.e.*, DCFH-DA, was used to stain the intracellular ROS, scale bar: 10 μm. Herein, Model means co-stimulation of RAW264.7 cells with LPS/IFN-γ for 24 h; and the groups (50 μg/mL and 100 μg/mL) mean co-incubation of RAW264.7 cells with PMPB NC with varied concentrations (50 μg/mL and 100 μg/mL) for 2 h, followed by co-stimulation with LPS/IFN-γ for 24 h. **b**–**d** The levels of some typical inflammatory cytokines secreted by RAW264.7 cells, *e.g.*, IL-6 (b), TNF-α (**c**) and MCP-1 (**d**), and they were detected by enzyme-linked immunosorbent assay (ELISA). Data are expressed as mean ± SD (n = 3), and *P˂0.05 and **P˂0.01, which were obtained in comparison to Model

Inspired by the excellent ROS scavenging ability of this biomimetic PMPB NC, anti-oxidation and anti-inflammation using PMPB NC can be anticipated. Cellular-level ROS depletion was supervised when incubating with PMPB NC. Before it, the cytotoxicity of PMPB NC on macrophages (RAW264.7 cells) was evaluated, and no evident toxicity is observed, suggesting the biosafety of PMPB NC (Additional file 1: Figure S9). More significantly, massive PMPB endocytosis by RAW264.7 cells also warrants the creditability of cellular-level evaluation (Additional file 1: Figure S10). The ROS indicator, 2,7-dichloro-dihydrofluorescien diacetate (DCFH-DA), was leveraged to monitor intracellular ROS variation in macrophages (RAW264.7 cells). Neglectable ROS production is detected in RAW264.7 cells alone (control) (Fig. 3a). Therefore, to guarantee the successful verification of cellular-level anti-oxidation and anti-inflammation abilities of PMPB, Model group was set, wherein RAW264.7 cells were co-stimulated by lipopolysaccharide (LPS)/IFN-γ to produce abundant ROS (Fig. 3a). Once incubating with PMPB NC, the ROS level was drastically dropped, as evidenced by the comparison between Model group and 50 µg/mL group (Fig. 3a), and more PMPB NCs bring about larger decreasing amplitude. This compelling result validates the strong anti-oxidation ability of PMPB NC.

It has been accepted that anti-inflammation is usually accompanied with anti-oxidation [7]. In this regard, PMPB NC is also expected to favor inflammation mitigation or decline. Inflammation-associated cytokine secretions (*e.g.*, TNF-α, IL-6 and MCP-1) were considerably repressed (Fig. 3b–d), which forcefully supports that PMPB NC features anti-inflammation. The distinctive functions (*i.e.*, anti-oxidation and anti-inflammation) of PMPB NC enable atherosclerotic plaque recession using PMPB NC.

### In vivo atherosclerosis treatment

Before in vivo treatment, the exploration of PMPB NC accumulation for localizing atherosclerosis is necessary. It is extensively accepted that detecting the early stage of atherosclerotic plaque and monitoring its evolution are of utmost importance because there are no evident symptoms until sever vessel occlusion or acute traumatic events that are arised from plaque shedding occur. Nevertheless, current detection modalities are limited to realize anatomy imaging for probing occlusion or hard calcification, but fail to track the biological evolution of atherosclerotic plaques [20].

**Fig. 4:**
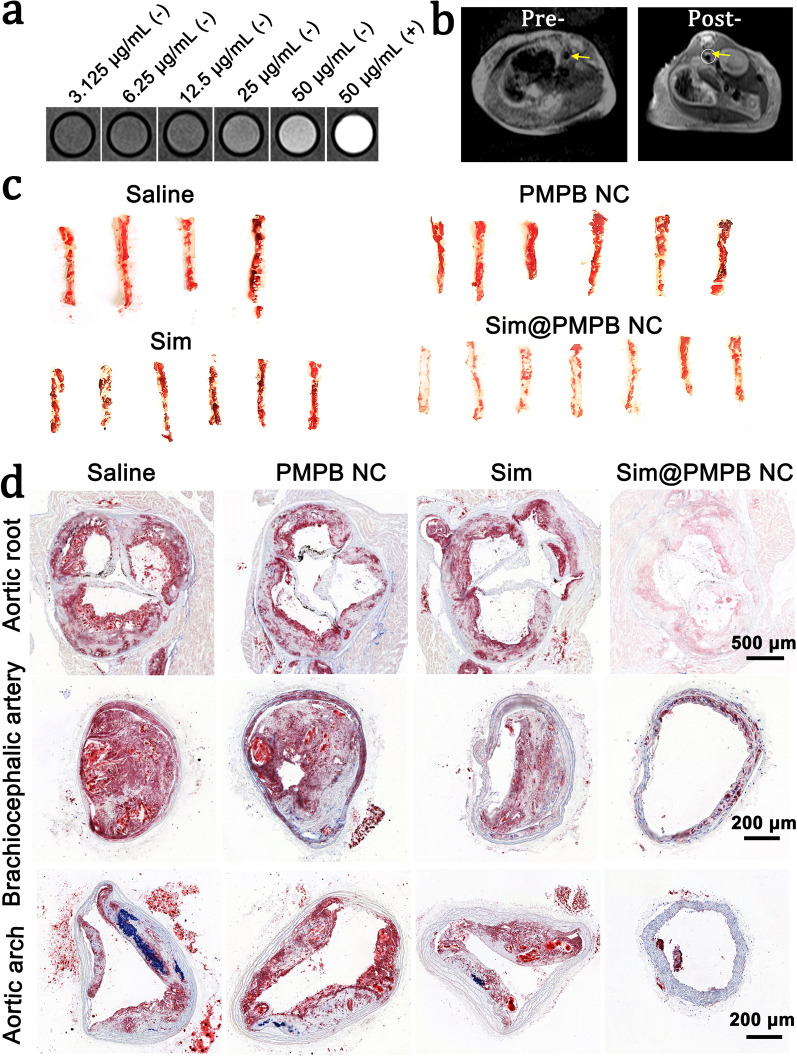
Theranostic effects of *i.v.* delivered PMPB NC in ApoE−/− mice. **a** In vitro T1-weighted MRI images of PMPB NC with varied concentrations under H_2_O_2_-free (−) and H_2_O_2_-included ( +) circumstances, and the H_2_O_2_ concentration was fixed at 20 µM. **b** In vivo T1-weighted MRI images of atherosclerosis in ApoE−/− mice under pre-injection or post-injection of PMPB NC. **c** Representative photographs of *en face* ORO-stained aortas from mice after treatment with different groups. **d** ORO-stained cryosections of the aortic root, aortic arch, and brachiocephalic artery. ApoE−/− mice were fed a cholesterol-rich and high-fat diet for 4 months. After the first month, different treatments were performed once a week within the last three months. Mice in the saline group were treated with saline alone, while other groups were separately administered with PMPB NC (20 mg/kg), Sim (2 mg/kg), and Sim@PMPB NC (20 mg/kg)

It has been well documented that Mn-based nanoparticles can be decomposed to generate Mn^2+^ in the presence of rich H_2_O_2_ in inflammation [29, 30], which enables Mn^2+^-enhanced T1-weighted MRI to magnify signals at desired diseases [40]. Inspired by this, Mn^2+^ release from PMPB NC responsible for illuminating the atherosclerosis can be expected, which also will benefit the monitoring of H_2_O_2_-associated treatment process. In vitro MRI results uncover that T1-weighted MRI signal intensity positively correlates with the concentration of PMPB NC in the absence of H_2_O_2_ (Fig. 4a and Additional file 1: Figure. S11). In contrast, once H_2_O_2_ is introduced, the MRI signal intensity was tremendously elevated. This appealing phenomenon can be attributed to H_2_O_2_-induced Mn^2+^ release from PMPB NC. Contributed by the Mn^2+^-enhanced MRI in atherosclerotic inflammation featuring rich H_2_O_2_, the atherosclerosis is entirely illuminated after intravenously injecting PMPB NC comparing to pre- (Fig. 4b), which lays a solid foundation to monitor H_2_O_2_-associated evolution of atherosclerosis.

On this account, in vivo atherosclerosis treatment deserves expectation especially uniting with Sim. To assess the treatment outcome, oil red O (ORO) was used to stain the harvested entire aortas. The largest ORO-stained region is observed in saline group (Fig. 4c). Contributed by ROS scavenging and inflammation alleviation by PMPB NC, PMPB NC exerts a treatment effect in decreasing plaques to some extent in comparison to saline. Of note, the decreased amplitude of ORO-positive area using PMPB NC is identical to that using the clinical drug (Sim). Intriguingly, a considerably-decreased red zones in aortas occurs to Sim@PMPB NC, meaning that the atherosclerotic plaques are subjected to highly-efficient treatment with Sim@PMPB NC.

To further identify them, ORO-stained cryosections of aortic sinus, brachiocephalic artery and aortic arch were examined. It is obviously found that PMPB NC triggers evident difference in lowering plaques in both aortic sinus and brachiocephalic artery, akin to Sim. Sim@PMPB NC is endowed with the highest treatment activity for making plaques recede (Fig. 4d). This phenomenon suggests that Sim@PMPB NC outweighs either Sim alone or Sim@PMPB NC alone in resisting atherosclerosis. Together, these systematic results decide that Sim@PMPB NC can effectively repress the progression of atherosclerosis via reducing plaque-covered regions, in line with the conclusion that the ORO-stained entire aortas made (Fig. 4c).

### Mechanism exploration

**Fig. 5:**
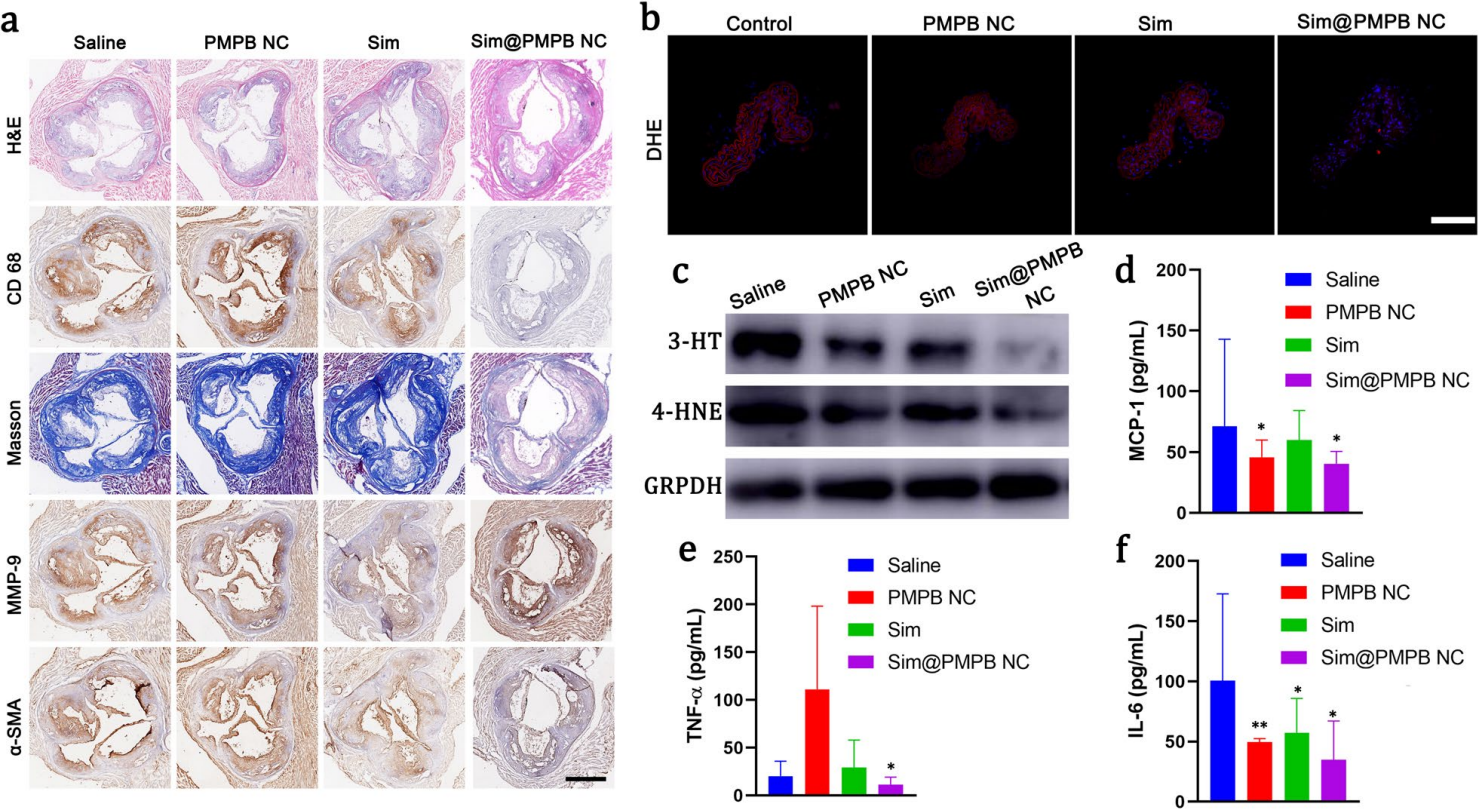
Pathological examinations and mechanistic explorations of Sim@PMPB NC treatment through analyzing oxidative stress and inflammation in ApoE−/− mice. **a** Representative immunohistochemical images of aortic root sections stained with H&E, CD68, MMP-9, Masson's trichrome, and α-SMA, and they were harvested from ApoE−/− mice that experienced a cholesterol-rich and high-fat diet for 3 months and different treatments (once a week) within the last two months. **b** Fluorescence images of DHE-stained sections of brachiocephalic artery after different treatments (control, PMPB NC, Sim, Sim@PMPB NC). **c**–**e** The levels of MCP-1 (**c**), IL-6 (**d**) and TNF-α (**e**) in serum of ApoE−/− mice after different treatments (control, PMPB NC, Sim, Sim@PMPB NC). **f** Western blotting bands of the representative expressions of 3-NT and 4-HNE. ApoE−/− mice were fed a cholesterol-rich and high-fat diet for 4 months. After the first month, different treatments were performed once a week within the last three months. Mice in the saline group were treated with saline alone, while other groups were separately administered with PMPB NC (20 mg/kg), Sim (2 mg/kg), and Sim@PMPB NC (20 mg/kg). Data are expressed as mean ± SD (n = 7), and *P˂0.05 and **P˂0.01, which were obtained in comparison to Saline. Anti-oxidant drug-loaded prussian blue analogues have been constructed to boycott atherosclerosis via scavenging reactive oxygen species, mitigating inflammation, decreasing collagen accumulation, reducing fibrous cap thickness, hampering macrophage infiltration, blockading foam cell birth and inhibiting lipid protein internalization and eventually depleting plaques.

After different corresponding treatments, pathological examinations of treated atherosclerotic plaques were implemented to directly visualize the structure variation of plaques in aortic sinus sections. After hematoxylin–eosin (H&E) and various immunohistochemical staining, it is found that a large number of acellular and lipid-rich necrotic cores emerge in the saline group (Fig. 5a). In contrast, the necrotic core range is considerably shrunk in Sim@PMPB NC group. After experiencing anti-CD68 and anti-matrix metalloproteinase-9 (MMP-9) staining, MMP 9 proteins and macrophages almost cover the whole horizon in the saline group, while Sim@PMPB NC brings about the largest drops of macrophage infiltration and MMP-9 expressions in plaques (Fig. 5a). Furthermore, both masson staining and α-smooth muscle actin (α-SMA) staining indicate that Sim@PMPB NC treatment hampers collagen accumulation in plaques, suppresses the proliferation of vascular smooth muscle cells (VSMC), and consequently decreases fibrous cap thickness (Fig. 5a). These results validate the excellent anti-atherosclerosis outcome of Sim@PMPB NC since VSMC proliferation is also one hallmark of atherosclerosis. It has been generally accepted that collagen, necrotic core region, macrophage infiltration, MMP-9 expression and VSCM proliferation in or surrounding atherosclerotic plaques positively correlate with plaque vulnerability [10]. Regarding this, it is obtained that Sim@PMPB NC therapy can effectively stabilize atherosclerotic plaques.

Preliminary in vitro explorations revealed that ROS scavenging and inflammation removal by PMPB NC took the responsibility for atherosclerosis therapy. To verify their role in in vivo treatment, a fluorescent probe, *i.e.*, dihydroethidium (DHE) that can reflect oxidative stress, was adopted to stain brachiocephalic artery harvested from ApoE−/− mice after experiencing different treatments (Fig. 5b). In control group, the red fluorescence illuminates the whole artery, while the red signals in both PMPB NC and Sim groups recede, suggesting the alleviation of oxidative stress. When combing PMPB NC with Sim, the Sim@PMPB NC treatment receives the largest removal of oxidative stress due to the approximately complete vanishment of red fluorescence, validating the potent ROS scavenging ability of Sim@PMPB NC. In line with immunofluorescence results, western blotting (WB) analysis also demonstrate that the expression levels of 3-HT and 4-HNE proteins associated with ROS level gradually decrease (Fig. 5c), and the lowest expression level in the Sim@PMPB group indicates the largest-degree of ROS exhaustion. Likewise, the in vivo anti-inflammation was inspected, where three pro-inflammatory cytokines (*e.g.*, MCP-1, IL-6 and TNF-α) in serum of ApoE−/− mice after different treatments were tested. Results show that these pro-inflammatory cytokines were significantly decreased by Sim@PMPB NC (Fig. 5d–f), validating Sim@PMPB NC can effectively attenuate inflammation in plaques.

To shed light on the underlying principle, a schematic that depicts the treatment process is provided in Scheme 1. Therein, Sim@PMPB NC eliminated systemic and local oxidative stress and inflammation, decreased macrophage infiltration and collagen/MMP-9 expressions in atherosclerotic plaques to stabilize plaques. These results will further result in the decreases of oxidized LDL internalization, foam cell formation and fibrous cap thickness, and all of which benefit atherosclerosis.

Ultimately, biosafety evaluation was carried out, and the weight of mice treated with Sim@PMPB NC almost keeps constant (Additional file 1: Figure S12). Blood analysis shows neglectable variations of routine blood and biochemistry indexes between control and Sim@PMPB NC with varied concentrations (Additional file 1: Figure S13). Additionally, the examinations of H&E-stained histological sections of major organs find no prominent injuries (Additional file 1: Figure S14). These results reflect the biosafety of Sim@PMPB NC. In particular, since PB nanoparticles have been approved by FDA, PMPB NC as one PB derivative is also believed safe. Therefore, the physical combination between PMPB NC and clinically-used Sim determines the high clinical translation potential of Sim@PMPB NC.

## Discussion

In summary, we successfully synthesized Sim-loaded theranostic agent based on PMPB NC (Sim@PMPB), where the PMPB carrier has been demonstrated to scavenge excessive ROS including free radicals and H_2_O_2_ and remove oxidative stress. Also, PMPB NC could hold up macrophage infiltration and appose pro-inflammatory cytokine secretions including (MCP-1, IL-6 and TNF-α). Contributed by the inspiring anti-oxidation and anti-inflammation of PMPB NC, PMPB NC exerted the anti-atherosclerosis activity to some extent. Especially, combining with Sim, the composite Sim@PMPB NC made the largest decreases of oxidative stress, pro-inflammatory cytokine secretion, macrophage infiltration, VSMC proliferation, collagen/MMP-9 expressions and fibrous cap thickness in atherosclerotic plaques in vivo. The marriage also considerably suppressed oxidized LDL internalization and foam cell formation, which determined that Sim@PMPB NC performed the best in stabilizing atherosclerotic plaques and alleviating atherosclerosis. Moreover, H_2_O_2_-induced Mn^2+^ release from PMPB NC was demonstrated to enhance MRI in atherosclerotic inflammation featuring rich H_2_O_2_, which is expected to monitor H_2_O_2_-associated evolution of atherosclerosis. Therefore, this biocompatible Sim@PMPB NC holds great potential in clinical translation for favoring atherosclerosis treatment.

## Methods

### Materials

K_4_[Fe(CN)_6_], bovine serum albumin(BSA), MnCl_2_, 2’,7’-dichlorofluorescin-diacetate (DCF-DA), Oil Red O (ORO), lipopolysaccharide (LPS), 2,2-diphenyl-1-picrylhydrazyl (DPPH), hydrogen peroxide assay kit (MAK 165) were purchased from Sigma-Aldrich (U.S.A.). 5,5-dimethyl-1-pyrroline-N-oxide (DMPO), 2,2,6,6-Tetramethylpiperidine 1-oxyl (TEMP) and simvastatin (Sim) were purchased from Sigma-Aldrich (Shanghai). (1,2-Distearoyl-sn-glycero-3-phosphoethanolamine-N-[methoxy(polyethylene glycol)-2000] (DSPE-PEG) was obtained from Corden Pharma (Switzerland). Cyanine5 NHS ester (Cy5) and cyanine7.5 NHS ester (Cy7.5) were from Lumiprobe (U.S.A.). Penicillin, streptomycin, fetal bovine serum (FBS), and Dulbecco’s Modified Eagle's Medium (DMEM) were purchased from Gibco (U.S.A.). 4’,6-Diamidino-2-phenylindole (DAPI), hematoxylin and 3,3’-dioctadecyloxacarbocyanine perchlorate (DiO) were purchased from Beyotime (China). LysoTracker Green was purchased from Invitrogen (U.S.A.). Recombinant murine interferon-γ (INF-γ) was purchased from PeproTech (U.S.A). Laser confocal scanning microscopy (CLSM)-specific dishes (35 mm × 10 mm) were purchased from Corning Inc (New York).

### Characterizations

Transmission electron microscopy (TEM) micrographs were obtained on JEM-2100F transmission electron microscope at 200 kV. Scanning electron microscopy (SEM) micrographs and corresponding element mapping were intuitively demonstrated on a field-emission Magellan 400 microscopy. Zeta potential and particle size distribution were measured on Zetasizer Nanoseries (Nano ZS90). UV − vis absorbance spectra recorded using an UV-3600 Shimadzu spectrometer. To further analyze chemical status of the samples, X-ray photoelectron spectra (XPS) analysis was performed on thermo ESCALAB 250 (Thermal Scientific, US). X-ray diffraction (XRD) patterns were measured on Rigaku D/MAX-2550 V XRD system. Electron spin resonance (ESR) was performed on Bruker-A3110-10/12 (Germany). FTIR spectra was characterized on Nicolet 7000-C spectrometer. Laser confocal scanning microscopy (LCSM) images were observed and recorded on Olympus FV1000 (Olympus Company). MR images were acquired on a 3.0 T clinical MRI scanner (Magnetom Verio TIM, Siemens Healthcare, Erlangen, Germany). N_2_ adsorption and desorption isotherms and pore diameter distribution were obtained on ASAP 2460 (Micromeritics Instrument Corp., USA).

### Synthesis of PMPB NC

K_4_[Fe(CN)_6_] (0.04 mmol) and BSA (50 mg) were dispersed into 10 mL of distilled water under magnetic stirring for 0.5 h, denoting as solution A. MnCl_2_ (0.05 mmol) and BSA (50 mg) were dispersed into 10 mL of deionized water distilled water under magnetic stirring for 0.5 h (denoted as solution B). Then, solution A was added slowly into solution B and stirred for another 1 h. The mixed solution was aged at room temperature for 24 h. The products were collected by centrifugation and washed with distill water for several times. Ultimately, PMPB NC was acquired after freeze drying.

### Constructions of Sim@ PMPB NC

Firstly, the obtained PMPB NC was dispersed in distill water, then the simvastatin (Sigma-Aldrich, Shanghai, China) (1 mg) was slowly added and stirred at room temperature (RT) for 12 h. Then, the mixture was centrifuged and washed with distill water several times, and the bottom products were collected. The Sim@PMPB NCs were obtained after freeze dying.

The scavenging performance of PMPB NC on ·OH by ESR using 5,5-dimethyl-1-pyrroline-N-oxide (DMPO) as capturing agents.

ESR with spin trap 5,5-dimethyl-1-pyrroline-N-oxide (DMPO) was selected to detect hydroxyl generation. The classical fenton reaction (FeSO_4_ + H_2_O_2_) was used to generate ·OH. Different concentrations of PMPB NC (0, 50, 25, 12.5 μg/mL) were added into the solution of FeSO_4_ + H_2_O_2_ with the concentration of 1 mg/mL and 50 mM DMPO for determining ·OH. As well, TiO_2_ was used to generate singlet oxygen under xenon light irradiation (300 W), after which different concentrations of PMPB NC (0, 50, 25, 12.5 μg/mL) were added as well as TEMP (50 mM).

### Determination of ROS-scavenging capability

The ROS-eliminating capability of PMPB NC was examined. To evaluate H_2_O_2_-scavenging capacity, different concentrations (from 0, 3.125, 6.25, 12.5, 25, 50, 100, to 200 μg/mL) of PMPB NC were incubated in 2.5 mL of 0.01 M PBS (pH 7.4) containing 500 mM H_2_O_2_ for 48 h. Then residual H_2_O_2_ was determined by a fluorimetric hydrogen peroxide assay kit (MAK165, Sigma-Aldrich), and eliminated H_2_O_2_ was calculated.

The ROS scavenging ability of PMPB NC was measured using a previously-established protocol. Briefly, 1 mL of a fresh solution of DPPH• (100 μg/mL) was incubated in 2 mL of methanol containing different concentrations of PMPB NC (from 0, 31.25, 62.5, 125, 250, to 500 μg/mL) for 30 min in the dark. Subsequently, the absorbance at 517 nm was recorded by UV–visible spectroscopy and eliminated DPPH was calculated.

### In vitro cytotoxicity assay

A mouse macrophage cell line (RAW264.7), obtained from Shanghai institute of Cells, Chinese Academy of Sciences, were cultured at 37 °C under 5% CO_2_ atmosphere. RAW264.7 cells were grown in Dulbecco’s Modified Eagle’s Medium (DMEM, high glucose, GIBCO, Invitrogen) with 10% fetal bovine serum (FBS) and 1% penicillin/streptomycin. For the cell viability assay, RAW264.7 cells were pre-seeded in 96-well plates at 1 × 10^4^ cells/well, which were further incubated with Sim@PMPB NC at varied concentrations for 24 and 48 h, respectively. Subsequently, the media were replaced with CCK-8 (100 μL, VCCK-8: VDMEM = 1: 9) and the cell viabilities were measured on a microplate reader at the wavelength of 450 nm after 60 min.

### Intracellular endocytosis of Sim@PMPB NC observation

RAW264.7 cells at a density of 1 × 10^5^ were seeded into the confocal laser scanning microscopy (CLSM)-specific dishes (35 × 10 mm, Corning Inc., New York) and incubated for 12 h. After that, the culture media was replaced by Sim@PMPB NC dispersion in DMEM (1 mL, 20 μg/mL in DMEM), and another three incubations for 2, 4 and 8 h, respectively, were carried on. Subsequently, the culture media were discarded and the DAPI solution (Beyotime Biotechnology) (VDAPI: Vmethanol = 1: 5) was added to stain cell nuclei. The CLSM observation was carried out on CLSM (FV1000, Olympus Company, Japan) after staining for 10 min.

### ROS-scavenging capability at cellular level

Four groups, *i.e.*, control, model, PMPB (50 μg/mL) and PMPB (100 μg/mL), were set to explore the ROS-scavenging capability of PMPB NC. RAW264.7 cells were cultured in 12-well plates for 12 h. Except control group, cells in other groups were stimulated with LPS (100 ng/mL) and IFN-γ (100 IU/mL). The cells in PMPB NC group were pretreated with PMPB NC for 2 h, and then stimulated with LPS/IFN-γ for 4 h. Those cells in control group were treated with fresh medium, and the cells in model group were stimulated with LPS/IFN-γ without treatment. Subsequently, the culture media were replaced by DCFH-DA solution (100 μ 1/9 μl in DMEM, Beyotime Biotechnology). Cells were rinsed and treated with DCFH-DA (10 μM) in serum-free DMEM. After another 30 min, the cells were finally rinsed with PBS for three times and then observed by CLSM.

### In vitro anti-inflammatory effects of PMPB NC in macrophages

RAW264.7 cells were seeded in 24-well plates at 1 × 10^5^ cells per well. The cells were divided into four groups as mentioned above. After 24 h, the cells were collected for Flow cytometry (FCM) analysis.

### Treatment of atherosclerosis in ApoE^−/−^ Mice with PMPB NC

ApoE^−/−^ mice were supplied from the Laboratory Animal Center of Shanghai Tenth People’s Hospital, and all experiments in vivo were performed according to protocols approved by the Laboratory Animal Center of Shanghai Tenth People’s Hospital and were in accordance with the policies of the National Ministry of Health. Mice were randomly assigned into four groups (n = 7), *i.e.*, Saline, PMPB NC, Sim and Sim@ PMPB NC. The mice were fed with a cholesterol-rich and high-fat diet for four months. After the first month, different treatments were performed once a week within the residual three months.

Detection of the atherosclerotic plaque after staining by ORO, H&E, Masson and other immunofluorescence or immumohistochemistry.

At the end of experimental period, all apoE^−/−^ mice were euthanized. In order to observe the extent of aortic lesion from left common carotid artery to iliac artery bifurcation, the aorta was excised and perfused with 10% neutral buffered formalin for 50 min. The aorta were opened longitudinally and stained with ORO or H&E or Masson, and then they were observed under the optical microscopy. As for DHE immunofluorescence staining, confocal fluorescence microscopy was used.

As for immumohistochemical staining, all apoE^−/−^ mice were euthanized at the end of experimental period. The aorta was excised and perfused with 10% neutral buffered formalin for 50 min. The aorta was opened longitudinally and co-stained with CD68 antiboody, MMP-9 antibody and α-SMA antibody, and then optical microscopic observation was carried out. Additionally, the fresh aorta in different groups was excised for WB analysis at the end of experimental period.

### In vivo tests of anti-inflammation and anti-oxidation

ApoE^−/−^ mice were randomly assigned into four groups (n = 7), *i.e.*, Saline, PMPB NC, Sim and Sim@ PMPB NC. The mice were fed with a cholesterol-rich and high-fat diet for four months. After the first month, different treatments were performed once a day for three times in total. After 6 h post-last treatment, all apoE^−/−^ mice were euthanized, and their serum were collected for ELISA analysis to determine pro-inflammatory cytokines (IL-6, TNF-α and MCP-1).

### Biosafety evaluation in vivo

All animal experiments were in agreement with the guidelines of the Regional Ethics Committee for Animal Experiments and the care regulations approved by the administrative committee of laboratory animals of Shanghai Tenth People’s Hospital. Healthy female Kunming mice (~ 18 g) were purchased and raised at Laboratory Animal Center, Shanghai Tenth People’s Hospital. Kunming mice were randomly divided into 4 groups (n = 4): (1) control group, (2) Sim@PMPB NC in PBS (5 mg kg^−1^), (3) Sim@PMPB NC in PBS (10 mg kg^−1^), (4) Sim@PMPB NC in PBS (20 mg kg ^− 1^). The body weight of mice was recorded every 2 days and scarified at the 28th day after intravenous administration of Sim@PMPB NC. Routine blood test including white blood cell (WBC), red blood cell (RBC), lymphocyte(LYM), creatinine (CR), mean corpusular volume(MCV),hemoglobin (HGB), hematocrit (HCT), mean corpuscular hemoglobin (MCH), blood urea nitrogen(BUN),mean corpuscular hemoglobin concentration (MCHC) and platelet count (PLT) were measured on Sysmex XS-800i automated hematology analyzer. Then, the major organs (heart, liver, spleen, lung, and kidney) were sectioned into slices and stained with hematoxylin–eosin staining (H&E) for histological analysis.

### Statistical analysis

Statistical analysis of all data (mean ± S.D) were performed using SPSS 22.0 (IBM Corp.). One-way analysis of variance and Chi-square test were used for the statistical evaluation. *P* < 0.05 was set as the statistical significance.

## Supplementary Information

**Additional file 1.** Additional figures.

## Data Availability

All data used to generate these results is available in the main text and supporting information.
