# Supplementary material for: Reactive oxygen species scavenging and inflammation mitigation enabled by biomimetic prussian blue analogues boycott atherosclerosis
Source: J Nanobiotechnology. 2021 May 31;19:161. doi: 10.1186/s12951-021-00897-2 (PMC8166117; doi:10.1186/s12951-021-00897-2)
Supplement: Supplementary file 1 — Additional file 1. Additional figures. [file 12951_2021_897_MOESM1_ESM.pdf]

Reactive Oxygen Species Scavenging and Inflammation Mitigation Enabled by Biomimetic Prussian Blue Analogues Boycott Atherosclerosis

Yan Zhang,[#,1] Yifei Yin,[#,1] Wei Zhang,[#,2] Hongyan Li,[1] Taixia Wang,[1] Haohao Yin,[1] Liping Sun,[*,1] Chunxia Su,[*,3] Kun Zhang[*,1] and Huixiong Xu[1]

Supplementary Figures

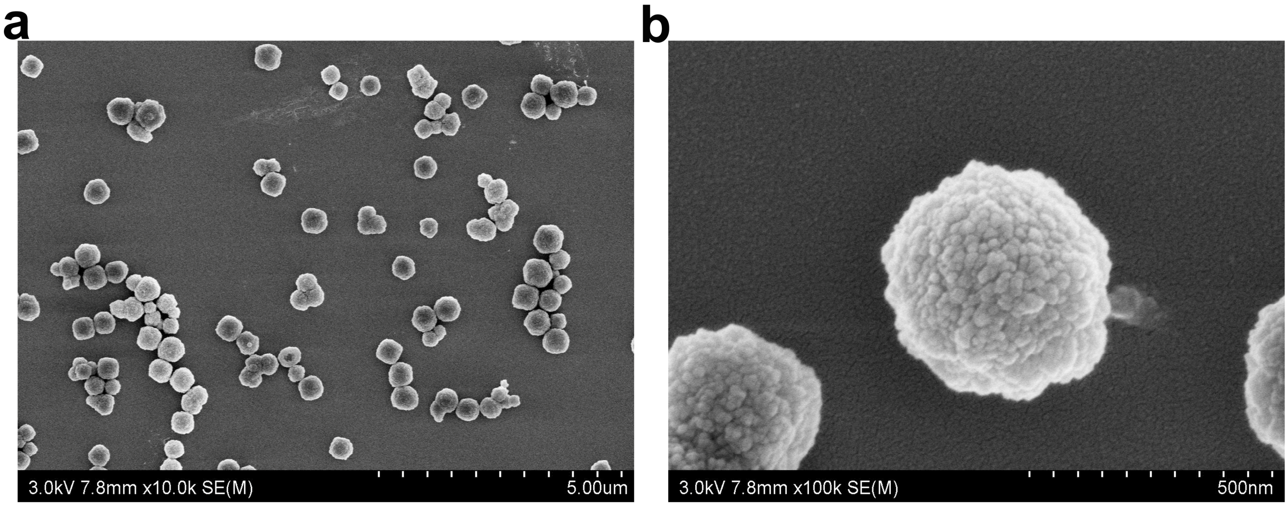

Figure S1 SEM images of PMPB NC at low (a) and high (b) magnification folds, respectively.

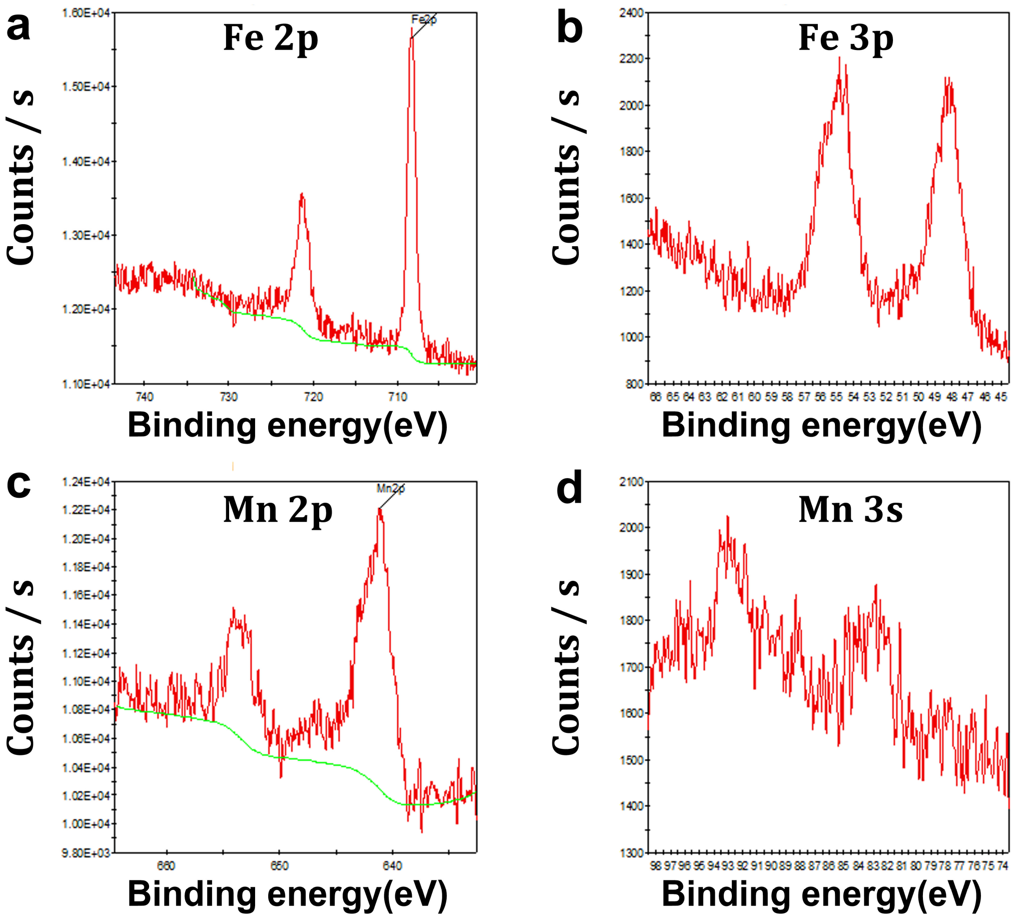

Figure S2 Narrow-window XPS spectra of Fe and Mn atoms in PMPB NC at different electron orbits: (a) Fe 2p, (b) Fe3p, (c) Mn 2p and (d) Mn 3s.

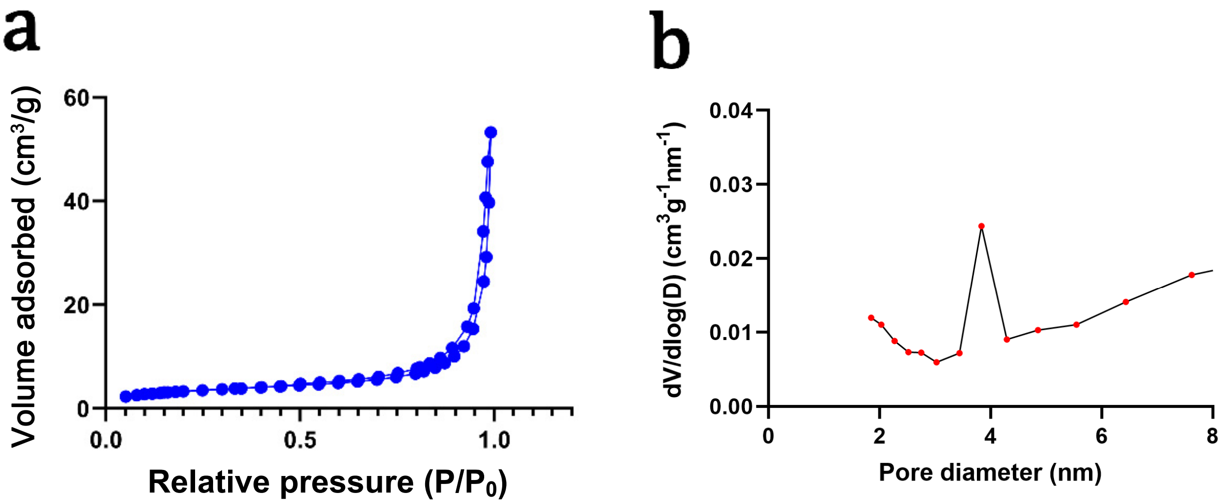

Figure S3 (a) N_2 adsorption and desorption isotherms of PMPB NC, and (b) Pore diameter distributions of PMPB NC.

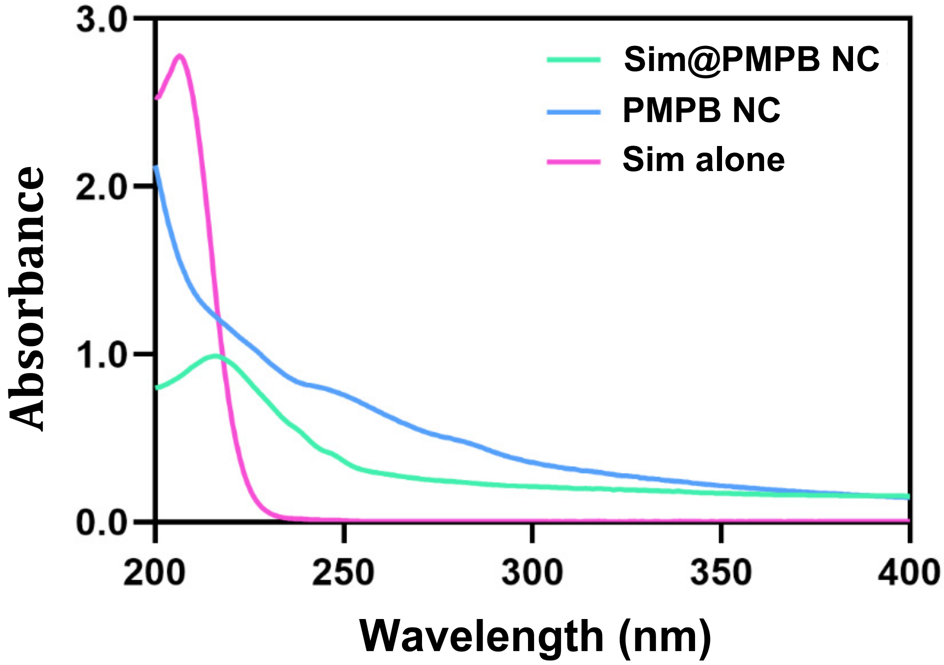

Figure S4 UV–vis spectra of free Sim alone, PMPB NC, Sim@ PMPB NC.

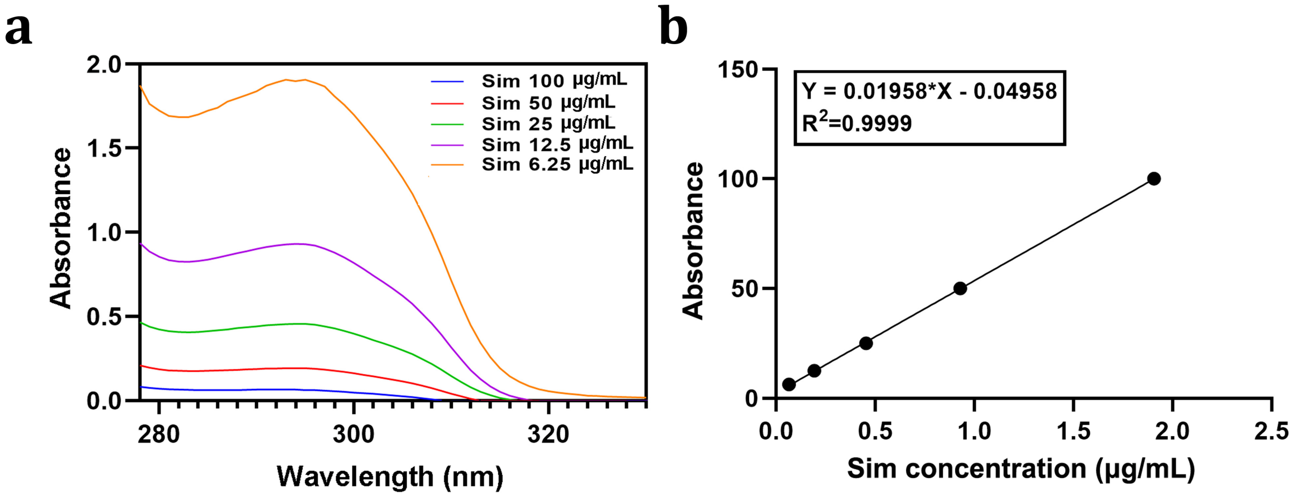

Figure S5 (a) UV–vis spectra of Sim at different concentrations. (b) Concentration-dependent standard curve of Sim that was obtained according to the absorbance intensity peaking at 294 nm from a.

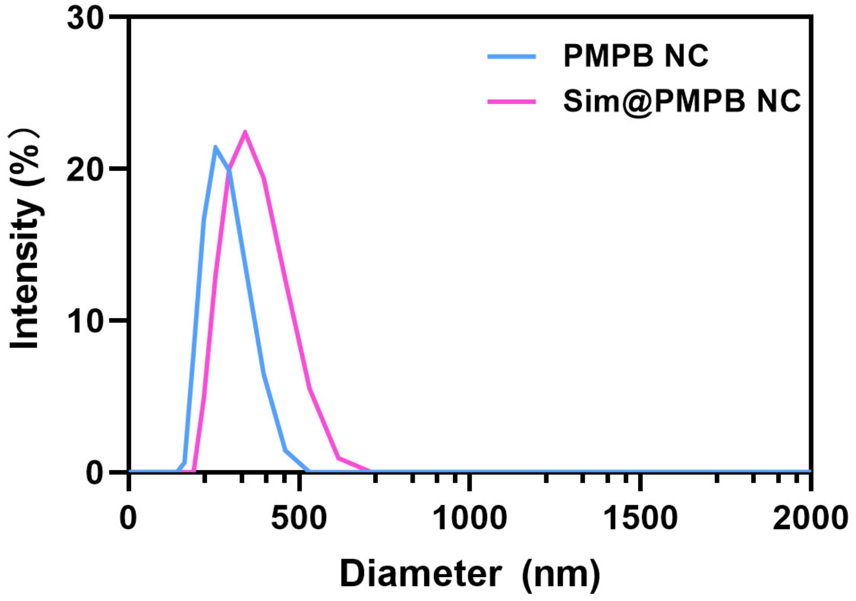

Figure S6 Hydrodynamic diameter distribution curves of PMPB NC and Sim@PMPB NC with polydispersity indexes: 0.189 and 0.231, respectively.

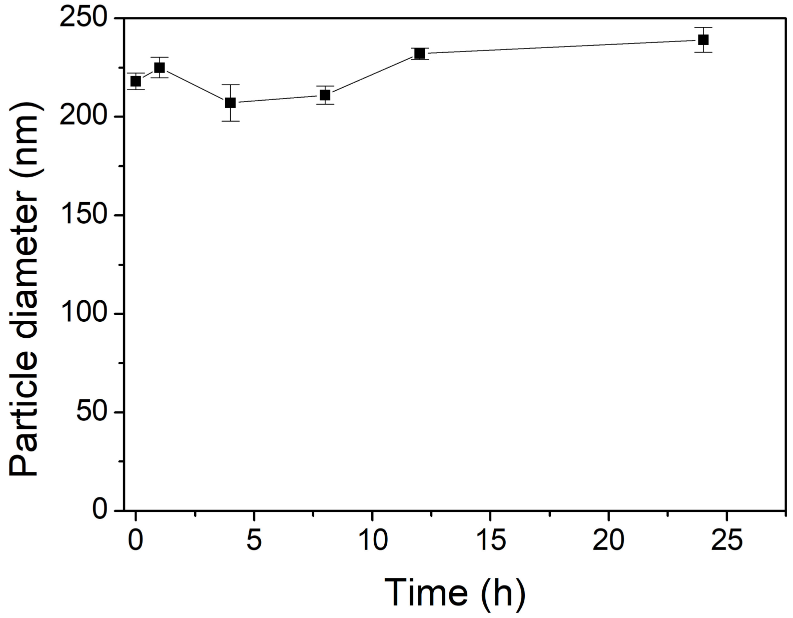

Figure S7 Time-dependent particle size of Sim@PMPB NC in PBS, which is available for evaluating colloidal stability. Data are expressed as mean ± standard deviation (SD) (n=3).

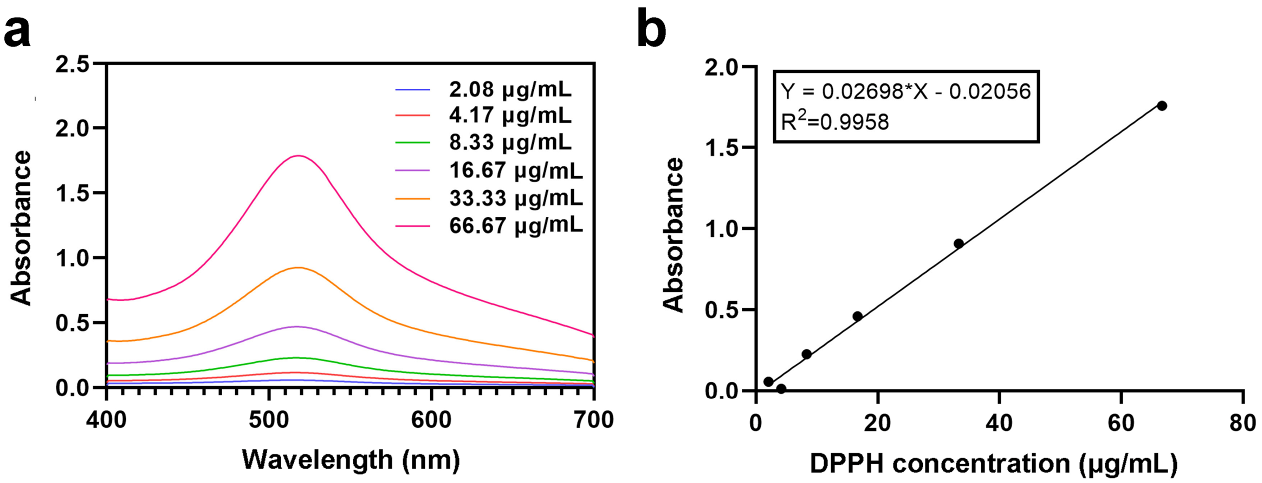

Figure S8 (a) UV–vis spectra of DPPH at different concentrations. (b) Concentration-dependent standard curve of DPPH that was obtained according to the absorbance intensity peaking at 525 nm from a.

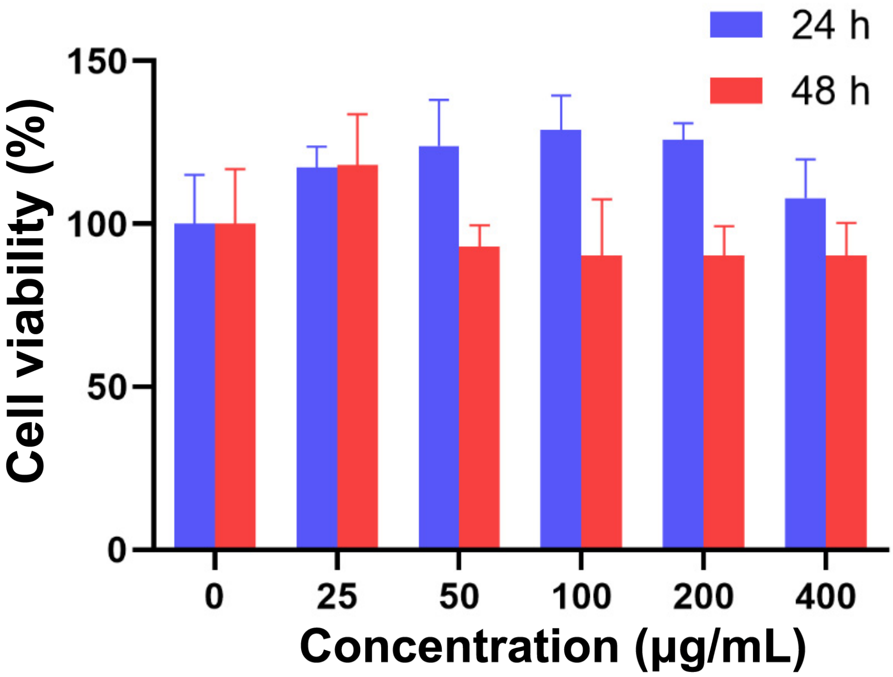

Figure S9 Cytotoxicity of Sim@PMPB NC after incubation with RAW 264.71 cells for 24 h and 48 h at varied mass concentrations (0, 25, 50, 100, 200, 400 μg/mL).

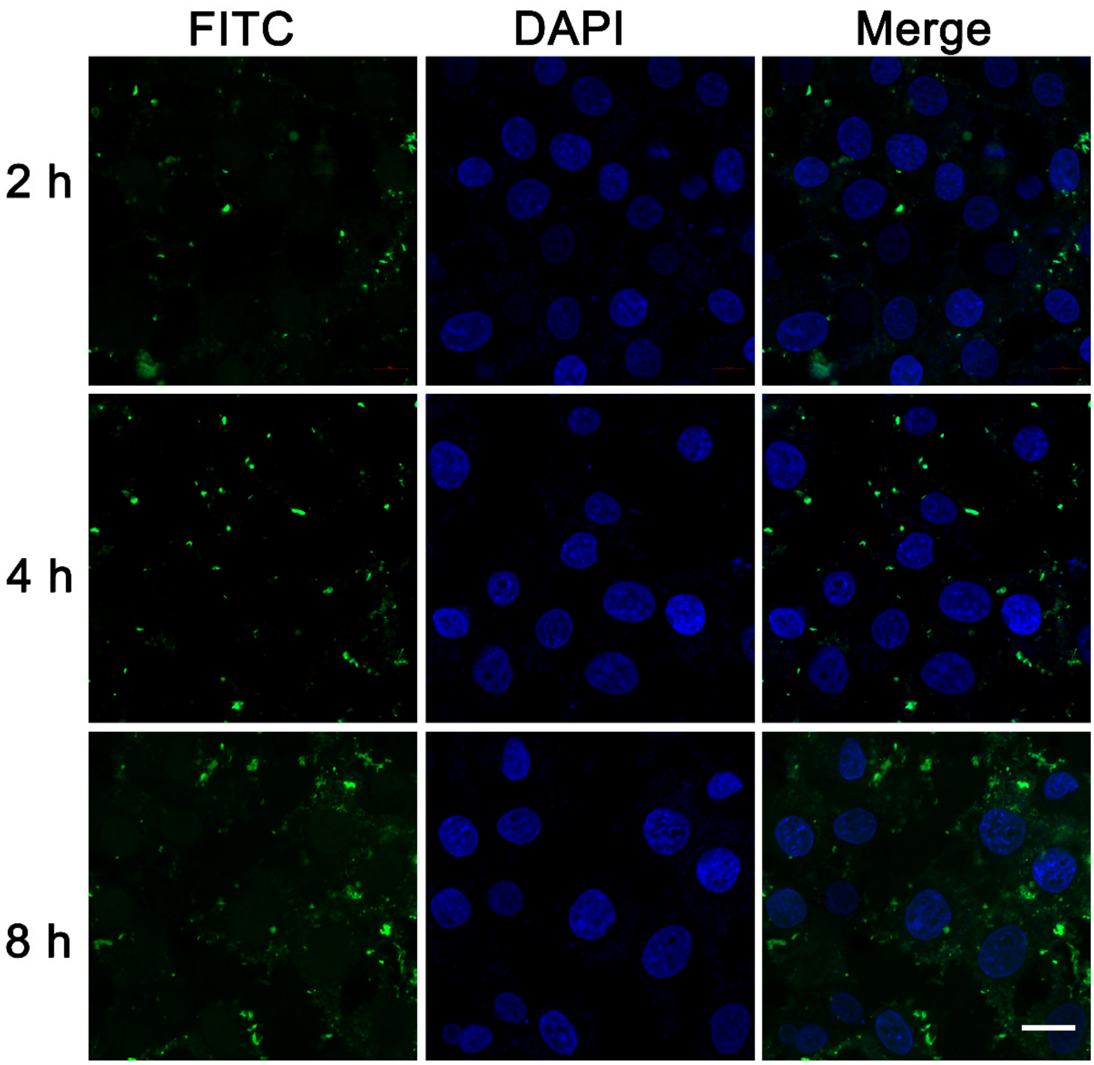

Figure S10 CLSM images of RAW264.7 cells after incubation with FITC-labeled Sim@PMPB NC for 2 h, 4 h and 8 h, respectively. The nuclei of RAW264.7 cells were stained by DAPI (blue) (Scale bar: 10 μm).

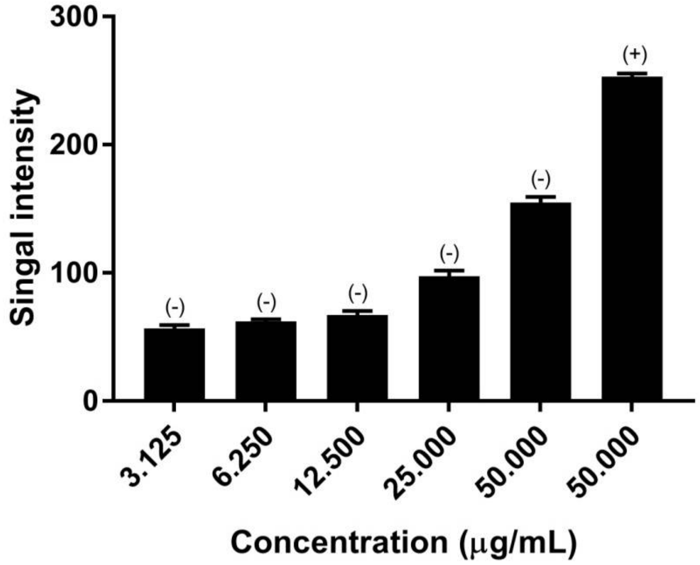

Figure S11 T1-weighted MRI signal of PMPB NC with varied concentrations, note. (-) and (+) represent H_2O_2-free and H_2O_2-included circumstances, respectively, and the H_2O_2 concentration was fixed at 20 μM. Data are expressed as mean ± SD (n=3).

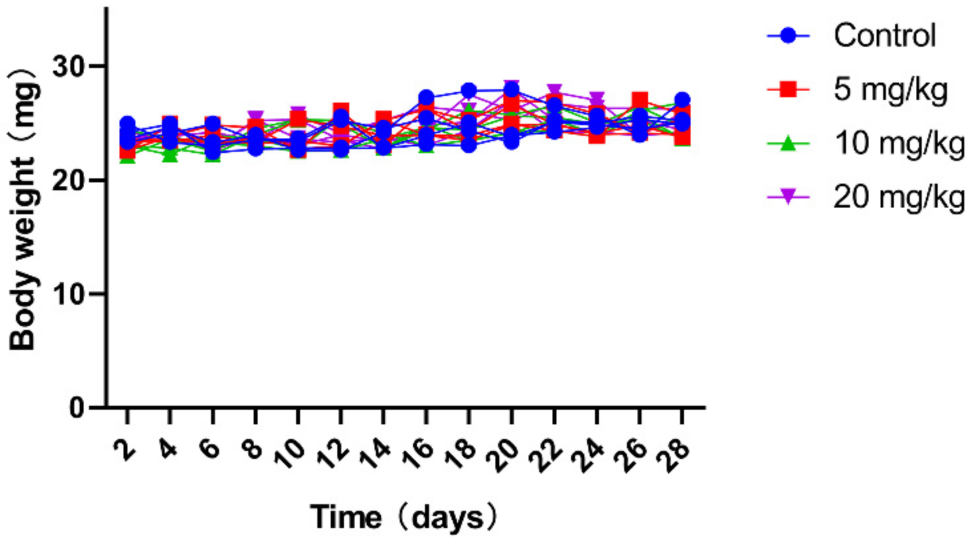

Figure S12 Time-dependent body weight curves of Kunming mice that experienced intravenous injection of Sim@PMPB NC with different concentrations (0 mg/kg, 5 mg/kg, 10 mg/kg and 20 mg/kg). Data were expressed as mean value ± SD (n=4).

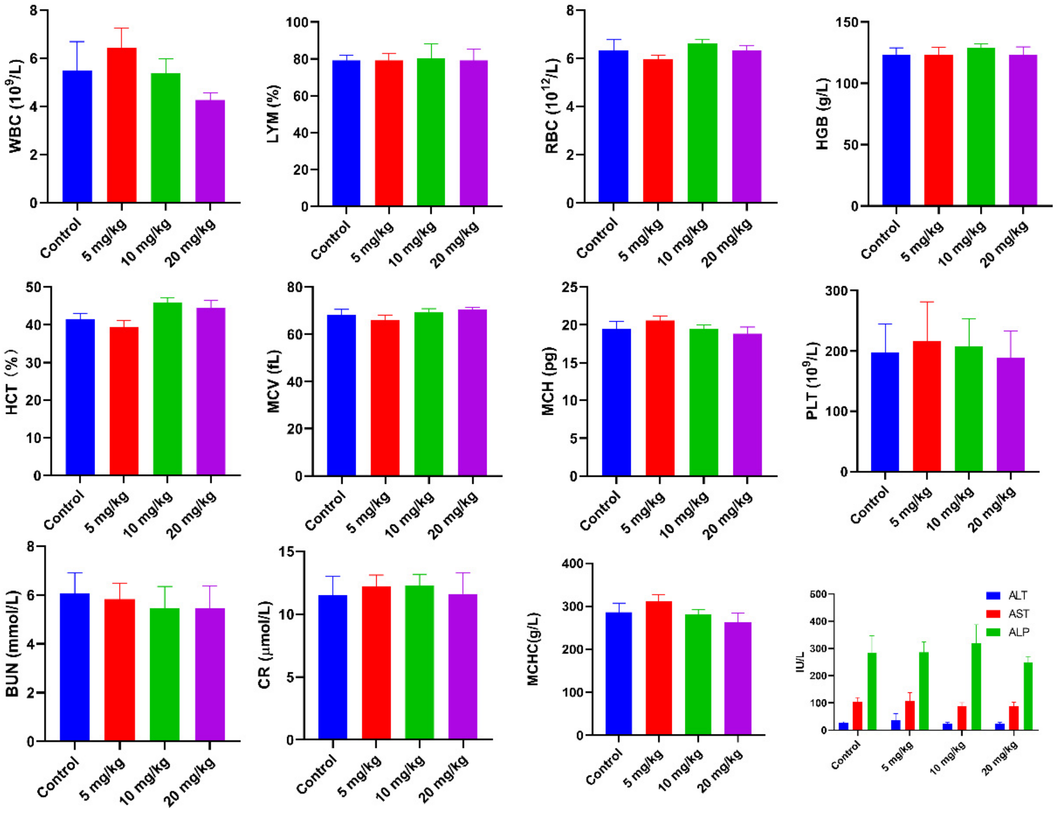

Figure S13 Blood biochemical indexes of mice in Control group and three other treated groups wherein Sim@PMPB NC with varied concentrations of 5 mg/kg, 10 mg/kg and 20 mg/kg were intravenously administrated into mice and another 28-days incubation was implemented. Data were expressed as mean value ± SD (n=4)

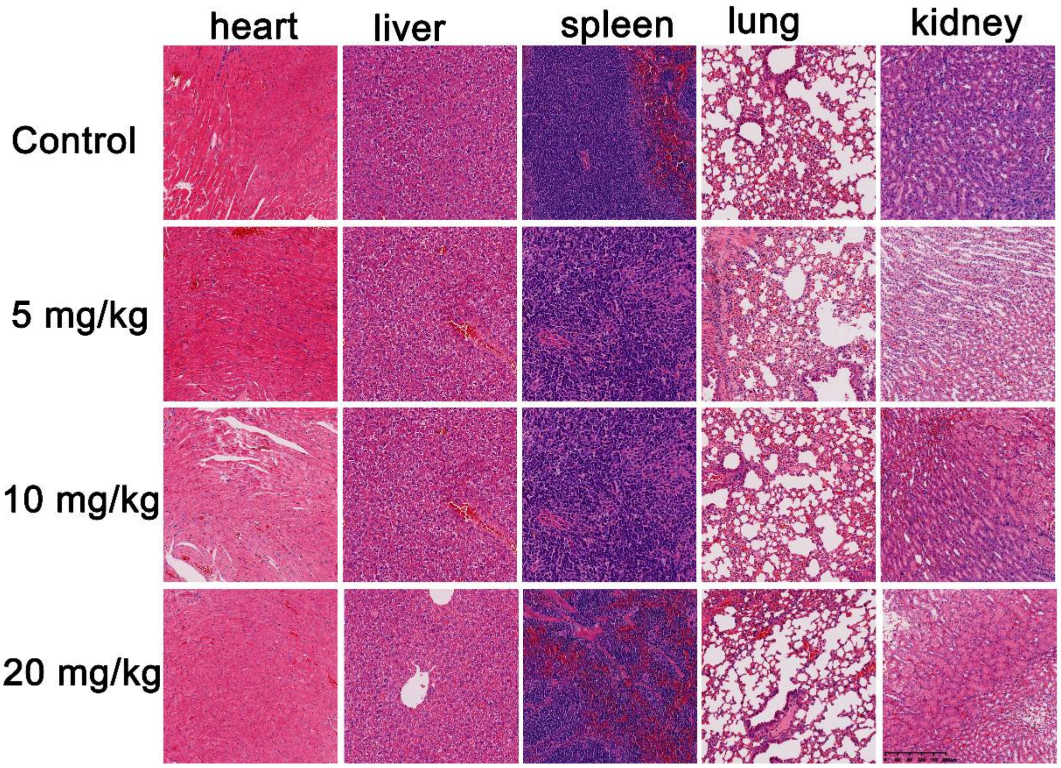

Figure S14 H&E staining microscopic images of normal organs (*i.e.*, heart, liver, spleen, lung and kidney) in mice that experienced *i.v.* injection of Sim@PMPB NC with varied concentrations (*i.e.*, 5 mg/kg, 10 mg/kg, 20 mg/kg) and another 28 days feeding. (Scale bar: 200 μm).
